# Qualitative Research on the Training Experiences and Needs of Intensive Care Unit General Nurses Against the Background of Regular Coronavirus Disease 2019 Prevention and Control

**DOI:** 10.3389/fpubh.2022.919987

**Published:** 2022-07-29

**Authors:** Yu Zhang, Yan-li Wang, Shu-qin Wang, Chun-yan Zhang, Na Wan, Yan-rui Jia, Feng-li Gao

**Affiliations:** ^1^Department of Respiratory and Critical Care Medicine, Beijing Institute of Respiratory Medicine, Beijing Chao-Yang Hospital, Capital Medical University, Beijing, China; ^2^Department of Surgical Intensive Care Unit, Beijing Chao-Yang Hospital, Capital Medical University, Beijing, China; ^3^Nursing Department, Beijing Chao-Yang Hospital, Capital Medical University, Beijing, China

**Keywords:** general nurses, qualitative research, coronavirus disease 2019, intensive care unit, training experiences

## Abstract

**Objective:**

This study aimed to investigate the training experiences and needs of intensive care unit (ICU) general nurses against a background of regular Coronavirus disease (COVID-19) prevention and control.

**Methods:**

Using the phenomenological method of qualitative research, semi-structured interviews were conducted with 10 ICU nurses. The interview data were analyzed, sorted, summarized, and refined using the content analysis method.

**Results:**

The following five themes were extracted from the interviews, based on the training experiences of the nurses: 1) broadening their thinking; 2) discovering their personal shortcomings; 3) gaining self-confidence; 4) calmly facing frontline work; 5) experiencing high assessment pressure. The training needs of the nurses could be summarized into the following four themes: 1) increased training time; 2) improving the assessment mechanism; 3) establishing a normal rotating-shift training system; 4) balancing the teaching levels.

**Conclusion:**

Against a background of regular epidemic prevention and control, ICU training for general nurses is of high practical significance and value. Thus, active exploration and research should be conducted to establish perfect training and assessment mechanisms for these nurses. Additionally, training methods that are suitable for clinical needs should be formulated and training systematization and standardization must be promoted.

## Introduction

The global Coronavirus disease 2019 (COVID-19) epidemic poses a major threat to public health worldwide (1). Currently, China's novel coronavirus pneumonia (NCP) epidemic prevention and control strategy reflects significant achievements and has entered a stage of regular COVID-19 prevention and control. The country has successfully implemented control strategies (2), with digital health as a preventive measure to support (3). Using this strict and large-scale mechanism, the number of NCP cases in China has been rapidly reduced. Nonetheless, there remains significant uncertainty about the epidemic situation (2).

In the initial stage of the disease, COVID-19 patients often develop influenza-like symptoms, with a large proportion rapidly developing severe acute lung injury, i.e., acute respiratory distress syndrome (4, 5). The treatment of severe COVID-19 patients often requires a variety of life support technologies, most importantly, extracorporeal membrane oxygenation (ECMO) technology, as recommended by the World Health Organization (6), which is a type of rescue treatment that may provide beneficial results when used by skilled clinical medical staff with ECMO management experience (7).

Intensive care unit (ICU) nurses are an important part of frontline medical teams and play a vital role in the treatment of patients with severe NCP. However, since ICU nurses have different specialties, they may experience anxiety as a result of having to cope with negative emotions in the context of engagement in specialties they are not particularly trained for (8–10). Therefore, to meet the urgent deployment needs of various public health emergencies, the nursing department of our hospital put forward the concept of ICU general nurses. The ICU general nurses refer to nurses from the ICU of one specialty, such as respiratory ICU, who has learned the relevant knowledge of the ICU of other specialties, including an emergency ICU, a cardiac surgical care unit, a surgical ICU, a coronary care unit, a neurosurgical care unit, etc. A detailed learning courses and plans are then developed based on the characteristics of each ICU and the shortcomings of nurse's specialized knowledge and skills. The program carried out four-week general knowledge and skills training (from February 27, 2021, to March 26, 2021), including a week-long theoretical course and 3 weeks of rotating ICU shifts within the hospital. The training content is shown in Table 1. A total of 16 nurses have to date completed this training and assessment.

**Table 1:** Theoretical course schedule (one week).

**Day**	**9:00–12:00**	**14:00–17:00**
1	Nursing of severe respiratory diseases (ARDS,COPD)	Atomization treatment
	Use of ventilator and alarm treatment	Vibration expectoration technique
2	Airway management	High-flow Nasal Cannula
	Prone ventilation	Postural drainage
3	Use of respiratory rehabilitation	Use of non-invasive ventilator
	Knowledge of hemodynamics	Lung auscultation
4	How to standardize enteral nutrition	Continuous renal replacement therapy (CRRT)
	Blood gas analysis	Mild hypothermia
5	PICCO connection, application and monitoring	Recognition of abnormal electrocardiogram
	Neurological assessment and intracranial pressure monitoring	The nursing of IABP

The current study adopted qualitative research methods to explore the training experiences and needs of ICU general nurses in the context of current regular epidemic prevention and control strategies to provide a reference for future explorations of the training mode for ICU nurses in general hospitals.

## Participants and Methods

### Study Design

A phenomenological and exploratory descriptive study approach was developed to investigate the training experiences and needs of ICU general nurses against the background of regular COVID-19 prevention and control.

### The Study Setting and Sample

The study participants were selected from a tertiary general hospital in Beijing. The nurses who participated in the general nurse training from six different ICUs, including a respiratory ICU, an emergency ICU, a cardiac surgical care unit, a surgical ICU, a coronary care unit, and a neurosurgical care unit.

To determine the sample size, the data provided by the respondents had to appear repeatedly until no new topics were found during the data analysis (data saturation). The researchers invited nurses who met the following inclusion criteria to participate in the study, i.e., all nurses who participated in the ICU general nurse training program and who agreed to participate in this study. While completing the sampling process, the researchers aimed to obtain diversified information related to the participants' demographic characteristics, such as gender, age, education level, professional title, and working years.

### Ethical Considerations

The participants provided signed informed consent for their inclusion in the study. The interviews were conducted following the participants volunteering to do so. Before conducting each interview, the researchers explained the purpose, significance, methods, and confidentiality principle of the study in detail and informed the participants that they had the right to withdraw from the study at any time without explanation. The Ethics Committee of Beijing Chao-Yang Hospital approved the study.

### Data Collection

The authors conducted individual semi-structured interviews in Chinese using a digital voice recorder to collect data and notes. The interviewers and the participants were colleagues in the same department and, accordingly, had existing harmonious relationships, which made the participating nurses more willing to express their true feelings. The participants were randomly assigned a letter (A–J) and were interviewed anonymously in alphabetical order, based on their assigned letter. The interviews were conducted in a conference room of the respiratory ICU of Beijing Chao-Yang Hospital.

The interviews took the form of open-ended questions and the gradual introduction of in-depth inquiries, based on the interviewees' answers. The interviews were conducted in a relaxed manner and the interviewees were listened to patiently without offering any guidance or suggestions. Each interview lasted between 30 and 40 min. The interviewers carefully observed any changes in the interviewees' expressions and emotions while recording their words, expressions, and movements in written form. All of the interviews were recorded. Once an interview was completed, each interviewee was asked to confirm the content of the written records to ensure the correctness of the recorded information.

The semi-structured interview included open-ended questions, such as the following:

(1) How did you feel/what was your experience like during the ICU general nurse training? Can you share these feelings with me? (2) What do you think of the curriculum, training content, and training methods used during the training? How would you evaluate their training effect? (3) What do you think of the teachers who delivered the training? Which subject left the biggest impression on you during the rotation, and why? (4) Do you think the ICU general nurse training will help you to reach the frontline of anti-epidemic work in the future? What will be your biggest gain from this training?

All of the study participants had more than 5 years of ICU experience. It is noted that when the participants' answers began reflecting repetition, the interviews were ended. This is known as “data saturation” because, at this point, no new information was contributed.

### Data Analysis

During the research process, the interviews were transcribed verbatim, and participants were invited to review and comment on their transcripts. We utilized Colaizzi's method to analyze the data (11). Our analysis team included two qualitative researchers. The audio recordings were transcribed verbatim by two researchers within 24 h of the interviews having been conducted, and additional observations of interviewees' non-verbal behaviors were added to the transcripts. Microsoft Word and Excel version 16.0 (Microsoft Corp, USA) were used for data management. The original transcripts and data analysis were recorded in Chinese; the researchers translated their content into English and explored linguistic and lexical nuances until all the researchers agreed with the results; this method helped to ensure that original meanings were retained.

By carefully reading and recapping the data, the researchers became wholly familiar with the content and gained an in-depth understanding of it. Next, they conducted manual hand analysis and extracted meaningful statements word-for-word, as well as important statements. The researchers developed general meanings for important statements until the two researchers reached a consensus. Next, the written expression of meaning was cut and pasted into a new table. The researchers arranged meanings into thematic clusters to find common important concepts that could serve as prototypes for the identified themes and described these in detail, defining each formerly identified theme by inserting three/four typical statements. Similar topics and descriptions were compiled and compared with extract common points of view. Finally, short and condensed phrases were used to describe each theme.

The two researchers then discussed the content of the categories. Revisions were made and a third researcher confirmed these. The three researchers then discussed the content until a consensus was reached. The categories were illustrated using quotes from the interviews. All three researchers were registered nurses, and one had a PhD and expertise related to quantitative and qualitative research methods. Finally, the training experiences and needs of ICU general nurses were extracted.

## Results

### Characteristics of the Participants

In this study, 10 nurses from six ICUs in class-three, Grade-A hospitals in Beijing (Beijing Chao-Yang Hospital, Capital Medical University, Beijing, China) were interviewed. Among them, eight were female, and two were male. Their ages ranged from 26 to 38 years, and their work experience in the ICU ranged from 5 to 16 years. There were eight senior nurses and two supervisor nurses. Seven had a junior college diploma, and three were undergraduates. The general information of the participants is shown in Table 2. The interview data were analyzed and refined, and the commonalities were identified. Then, two backbone tree nodes and nine themes of the ICU general nurses' training experiences and needs were summarized. Five themes were refined from their training experiences, and four themes were refined from their training needs.

**Table 2:** General characteristics of the interviewed nurses.

**Number**	**Gender**	**Age (years)**	**ICU experience (years)**	**Title**	**Education**
A	Male	36	15	Supervisor nurse	Junior college
B	Female	28	6	Senior nurse	Bachelor
C	Female	29	8	Senior nurse	Junior college
D	Female	38	16	Supervisor nurse	Bachelor
E	Male	33	9	Senior nurse	Junior college
F	Female	26	5	Senior nurse	Bachelor
G	Female	34	13	Senior nurse	Junior college
H	Female	35	14	Supervisor nurse	Junior college
I	Female	27	6	Senior nurse	Junior college
J	Female	32	10	Senior nurse	Junior college

*1. Age was calculated as the integer value based on date or birth*.

*2. In China, the Junior College Degree is three years and no degree can be obtained, while the undergraduate degree is four years and a Bachelor's Degree can be obtained*.

### Themes

Through in-depth analysis and the extraction of interview data, common themes and subthemes were identified regarding the training experiences and needs of ICU general nurses against the background of regular COVID-19 prevention and control. These data are summarized in Table 3.

**Table 3:** Training experiences and needs of ICU general nurses against the background of normalized COVID-19 prevention and control.

**Training experiences**	**Training needs and suggestions**
• Broadening their thinking	• Increasing the training time
• Discovering their own shortcomings	• Improving the assessment mechanism
• Gaining self-confidence	• Building a normalized rotating-shift training system
• Calmly facing front-line work	• Balancing the teaching level
• Experiencing high assessment stress	

### The Training Experiences of Intensive Care Unit General Nurses Against the Background of Regular Epidemic Prevention and Control

#### Broadening Thinking Processes

Three interviewees stated that preliminary theoretical courses had broadened their thinking, increased their understanding, and enriched their knowledge reserves. Following rotation in various ICUs after clinical practice training under the guidance of their teachers, nurses were able to apply in-depth considerations related to finding solutions to problems in their work. The following information was taken from the conducted interviews.

Nurse B: “In the SICU training, we learned about blood filtration and enteral nutrition. Before this training, I only knew how to do the procedures, but I did not understand the details of how they worked. The teachers explained this in great detail. They trained the nurses…how to conduct this blood filtration. You know the way to think about what you encounter in clinical practice. We learned about oxygen therapy in RICU, about blood filtration in SICU, and about mild hypothermia in EICU. As a result, we all gained a better understanding of the concept that the patient is not a single individual. Nurses who practice in the ICU of their own hospital will be reminded of the concepts they learned there. Unlike before, we only consider a single point.”

Nurse G: “After finishing the training, when you return to your ICU and the doctor gives you a ventilator parameter, you may question their instructions. For example, *is this correct? Why is it different from what I have learned in the respiratory monitoring class*?”

Nurse A: “Before attending the ICU general nurse training, we could not operate oxygen therapy in a standardized manner. For example, we could not manage airbags properly. Following the Artificial airway management training, however, when I go back, I will pay attention to all of these details. Additionally, in my practice, I will always ask myself, “Is it reasonable to do a procedure like this?”

#### Discovering Their Shortcomings

The interviewees believed that the training of ICU General Nurses would allow them to gain new knowledge and skills while concurrently identifying their shortcomings concerning knowledge of critical care nursing and departmental management in their daily work.

Nurse F: “I…learned a lot during surgical monitoring about aspects that I did not understand before.”

Nurse H: “I struggled a lot when…learning new information about the heart. All of the nurses found the topic of the heart difficult. For example, completing an electrocardiogram was difficult, and we had little knowledge about cardiac diseases.”

Nurse J: “What impressed me most was the surgical monitoring. The doctors and nurses were able to cooperate very well. Nurses can learn a lot from this process.”

#### Gaining Self-Confidence

In the 3 weeks of clinical rotation shifts, the ICU general nurses experienced continuous improvement and gained self-confidence by observing and learning how to use the clinical technology of each ICU specialty.

Nurse A: “I learned about many things I had not experienced before. I now have more confidence.”

Nurse C: “When we can meet the patients with different kinds of diseases, I am generally able to understand and master much better than before. I will thus be less confused and know what to pay attention to when I see them.”

Nurse D: “I learned a lot about aspects that are not evident in our department. As a result, we have better general knowledge and can better master what our department doesn't have. It mainly includes ventilator and blood filter which are seldom used in our department.”

Nurse F: “General education means learning something about everything. Next time, when I operate a new machine, for example, a new model or brand of hemofiltration machine, or a ventilator. I will be able to do so professionally.”

#### Calmly Facing Frontline Work

The training reduced to some extent the tension and stress nurses experienced due to a lack of knowledge and skills. When facing major public health events in the future, the ICU general nurses will be able to participate in frontline treatment work more calmly and confidently.

Nurse J: “The training will help me significantly in terms of participating in frontline nursing in the future. Having more general knowledge will help me to be more confident in the treatment and nursing of COVID-19 patients.”

Nurse H: “Through the observation and training I experienced in different ICUs, I became familiar with different working environments. In the future, when I assist other ICUs, I will be less anxious about practicing in a different environment.”

#### Experiencing High Assessment Stress

The interviewees believed that the training had been comprehensive but very short. Within 1 week, they had to finish an answer sheet of theoretical knowledge for each ICU specialty, prepare a PowerPoint presentation for delivering a short lecture, and pass an operational test. This was very stressful and required high-demanding trainees.

Nurse C: “I experienced a lot of pressure. Dealing with our daily nursing tasks was already very stressful. We did not need to make PowerPoint or lectures while working in our own hospitals. Preparing a presentation in such a short time was very difficult for me.”

Nurse E: “The difficulty of this assessment was that we had to prepare the presentation very quickly. I…revised the PPT at least 10 times.”

Nurse F: “I did not go home for a week to prepare for this exam. I reviewed the work in our department in the evenings.”

### The Training Needs and Suggestions Presented by Intensive Care Unit General Nurses

#### Increasing the Training Time

The four-week ICU general knowledge and skills training period included a one-week theoretical course and 3 weeks of ICU rotational shifts in the hospital. Each nurse was assigned to three ICUs for the shift rotations, working in one ICU per week. The nurses believed that the theoretical training and clinical practice times in each ICU had been too short. It is thus suggested that the training time be increased in the future to achieve better learning outcomes.

Nurse F: “One week in a department is very short…we cannot become proficient during such a limited time. For example, respiratory machines are complicated; even learning the connections of different tubes can take up to three days to figure out.”

Nurse J: “We should spend at least 1 month in a department and take care of patients according to the rotating shift schedule. If we can apply what we have learned to clinical nursing, we will be able to better retain these skills.”

Nurse H: “Three weeks is not enough for us to gain an in-depth understanding of all the different specialties. It takes at least half a year to master all of them, with 3 weeks spent in each department.”

#### Improving the Assessment Mechanism

The interviewees believed that the training placed too much emphasis on assessment. They expressed having to focus too much on the review and achievements of the assessment, which affected their learning to some extent. Thus, it is suggested that in the future, teachers should increase the training time, adjust the assessment form, and assess trainees at different stages.

Nurse G: “The training was very rewarding. However, it still requires some changes to allow trainees to maintain a balance between assessment and study. For example, due to anxiety about failing the exam we spent a lot of time…reviewing and reciting the questions while studying. By paying less attention to the assessment, we will be able to retain more knowledge.”

Nurse E: “For the degree of absorption of knowledge, I think I can accept 70–80%, but in fact, I only accept 40–50%. We may not be able to learn both theory and procedural knowledge while preparing for small lectures.”

Nurse I: “To conduct assessments…departments can assess training individually. For example, assessment can be conducted when the trainees finish learning from a specific department. The trainees can select one of the following three items as test topics, i.e., small lectures, theory, and procedures. In this way, when nurses are taking a class, they can focus their attention on the specific knowledge pertaining to this class. Furthermore, when taking a test, they can concentrate on reviewing the knowledge related to a specific department. The assessment should follow the training completed for each department. Nurses cannot manage all the assessments at once. Sometimes, to evaluate the learning effect of a certain content, an exam or a report is enough.”

#### Building a Regular Shift Rotation Training System

The interviewees considered the training to have been a positive experience. In the future, the training mechanism can be improved and the routine training of ICU general nurses should be promoted.

Nurse J: “The original intention of this training is very good. It introduces nurses to new modes of nursing. In the future, this training should be incorporated into a routine schedule similar to regular training. All ICU nurses regardless of years of experience should complete this course. They should participate in rotating-shift learning in each department and focus on when to listen…and what specifically to learn, based on their undergraduate course.”

Nurse H: “When we go back to our department, we'll do our job, just like before. My knowledge is still superficial. Therefore, this training should be regular. It cannot end at a specific point; instead, there must be a regular rotating-shift mechanism in place. Otherwise, only a small number of nurses will master general nursing knowledge because only those nurses learned.”

#### Balancing the Teaching Level

According to the respondents, nurses who have been in charge of teaching in the department for a long time have to be transferred from their current positions to support the front line of the epidemic response. The teaching level of temporary substitute teachers was observed as having been uneven; accordingly, more teachers must be trained to promote the effective development of general ICU training.

Nurse A: “The training teachers had different skill levels. Some teachers had limited abilities, and some were more knowledgeable. Thus, the teaching effect, to some extent, depended on the training teachers.”

Nurse C: “Many senior teachers had been involved in supporting frontline healthcare. The current teachers are less-experienced.”

## Discussion

### The Training of Intensive Care Unit General Nurses Is of Great Significance to Reserving Rescue Personnel for Public Health Emergencies

The global COVID-19 pandemic was listed as a public health emergency of international concern on January 30, 2020 (12). In China, a total of 42,000 medical staff, including 28,600 nurses, were sent to Hubei to support patients with COVID-19 (13). Patients with severe COVID-19 require disease observation, specimen collection, treatment plan implementation, and vigilant nursing. Thus, ICU nurses play an important role in the frontline of COVID-19 control, based on their professional skills. They need to closely monitor patients' vital signs, manage artificial airways, deliver renal replacement therapy, ECMO nursing, and prone ventilation, and actively prevent complications, such as ventilator-associated pneumonia, catheter-related blood flow infection, deep vein thrombosis, and pressure injuries (14).

In 1982, Benner first proposed the concept of general nursing ability, which was defined as “the ability of nurses to complete their work and achieve the desired results in a complex and changeable environment” (15). In 2003, after conducting research in many countries, the International Council of Nurses defined nurses' general nursing aptitude as “the ability of an individual to combine and effectively use knowledge, skills, and judgment in their daily work, which reflects the knowledge, understanding and judgment, cognition, skills, personal characteristics and attitude required by nurses to complete their basic work responsibilities” (16). However, the concept of ICU general nurses has, to date, not been clearly defined.

In public health emergencies, a nursing team comprising nurses from various ICU specialties can fully apply their professional skills and better undertake rescue operations. A hospital survey revealed that 66 (47.8%) of 138 ICU nurses surveyed had participated in the treatment of public health emergencies, suggesting that these nurses had been exposed to more practical opportunities for dealing with these emergencies (17). As the backbone of the emergency medical service system, ICU professional nurses assume the important task of delivering rescue operations. With the development of the nursing discipline, the specialty division of the ICU is becoming increasingly detailed to include specialized units, such as emergency, cardiac, respiratory, neurological, and other dedicated ICUs. Nurses have rich specialized knowledge and excellent technical skills in their own departments but may be relatively weak in terms of mastering the knowledge and skills of other ICU specialties (18). A training plan for ICU general nurses was carried out by our hospital to realize a combination of general and special/cultivated and comprehensive ICU nursing talents. This training will have a positive role in promoting China's medical rescue services and will be of significant aid in reserving rescue personnel for public health emergencies in the future. These aims were verified by the results of the present study. Most nurses who participated in the ICU general training stated that they had become more aware of their own shortcomings and, accordingly, were able to broaden their knowledge (10). When participating in future major public health events, these nurses will be able to more calmly and confidently contribute to frontline treatment. Additionally, the training reduced the tension and stress they experienced to some degree brought on by a lack of knowledge and skills.

### The Training Mode of Intensive Care Unit General Nurses Must Be Further Explored

As a new notion related to nursing talent training, ICU general nurses have practical significance in the current healthcare context. The advent of general nurse training can broaden the nursing training mode by combining specialty and general nursing via specialist nurse training. This training can ensure the reservation of “ICU general nurses” for frontline intensive COVID-19 care and can also be used for ICU nurses in general hospitals. Guan et al. (19) formed a multidisciplinary nursing team by combining general with specialized nursing to better meet the needs of disaster rescue operations.

Training general nurses is a current development trend in hospitals. This approach not only meets the needs of comprehensive nursing technology for critically ill patients but also creates a reserve of skilled personnel for emergency rescues. Intensive care unit general nurse training is still in the experimental stage. Based on the results of this study, the total training time and the time of each ICU practice stage were relatively short, and the assessment proportion was intense. From the observations conducted herein, nurses who participated in ICU general nurse training could not adapt to the density of training or the assessment mechanism. In the future, based on increasing the training time, this type of training should be promoted, and more practice stages should be developed to explore a scientific and systematic training mechanism in terms of the curriculum, training content arrangement, training methods, teachers, and assessment methods. Additionally, the training mode of ICU general nurses should be incorporated into the regular training mechanism, and general nursing lead teachers should gradually be assigned to ensure the stability of the teaching level. The current researchers aim to continue studying and exploring various methods of training and management that combine healthcare talents in public health-event rescue and nursing.

### Using Diversified Training Programs to Improve Intensive Care Unit Nurses' General Knowledge and Skills

Intensive care unit nurses being able to quickly respond and treat severely compromised patients in major public health events is key to ensuring the successful completion of rescue tasks in public health emergencies (8). The increasing demand for nurses in critical care brought on by the COVID-19 pandemic is significant (20). Specialist nurses are needed in the area of respiratory care, ventilator care, critical care, nursing management, and infection control (21). Therefore, cultivating standardized ICU nurses with strong emergency abilities through various platforms and multiple channels is important. This type of training should be proposed and regularly updated within the ICU knowledge base. Open training courses and desktop drills should be carried out regularly as on-the-job training for nurses. Additionally, controlled environments, such as workshops and simulated scenarios, should be provided for nurses to practice their skills (22). In the ICU nurse training program, respiratory, surgical, emergency, and other ICU departments should be established with training plans incorporated for nurses at different levels (23), as well as online courses that are based on mobile platforms to realize flexibility within the education system (21).

### Contributions and Limitations

The training of ICU general nurses is of great significance to improve the response ability of nurses to major health emergencies and the treatment ability of severe patients. The training is arranged in the stage of regular COVID-19 prevention and control, which is conducive to the establishment of talent echelon. ICU nurses looked forward to the training at the beginning, and were anxious about assessment during the training, and finally put forward a better research direction for our future research.

There are some limitations to the present study. First, the sample size is relatively small. Large-scale studies need to be performed to validate the current findings. Secondly, after the interview, the nurses returned to their ICU to continue their original work without further follow-up, which is also a limitation.

## Conclusion

The present study presented the experience of ICU nurses who participated in ICU general nurse training from five perspectives, i.e., broadening their thinking, discovering their skills deficiencies, gaining self-confidence, calmly facing frontline healthcare work, and managing assessment pressure. The training requirements were mainly reflected through the need for increased training time, improving the assessment mechanism, building a regular rotation training system, and balancing the teaching levels. By identifying these experiences and needs, this paper provided preliminary evidence for the future development of ICU general nurses as a method for enabling a better response to infectious disease emergencies, such as COVID-19.

## Data Availability Statement

The original contributions presented in the study are included in the article/supplementary material, further inquiries can be directed to the corresponding author/s.

## Ethics Statement

The studies involving human participants were reviewed and approved by Beijing Chao-Yang Hospital, Capital Medical University Ethics Committee. The patients/participants provided their written informed consent to participate in this study.

## Author Contributions

YZ and Y-rJ: conception and design of the research. YZ, Y-lW, and NW: acquisition of data. YZ and S-qW: analysis and interpretation of the data and writing of the manuscript. C-yZ: statistical analysis. F-lG: critical revision of the manuscript for intellectual content. All authors read and approved the final draft.

## Conflict of Interest

The authors declare that the research was conducted in the absence of any commercial or financial relationships that could be construed as a potential conflict of interest.

## Publisher's Note

All claims expressed in this article are solely those of the authors and do not necessarily represent those of their affiliated organizations, or those of the publisher, the editors and the reviewers. Any product that may be evaluated in this article, or claim that may be made by its manufacturer, is not guaranteed or endorsed by the publisher.

## Abbreviations

ICU: intensive care unit
ECMO: extracorporeal membrane oxygenation
ARDS: acute respiratory distress syndrome
COPD: chronic obstructive pulmonary disease
CRRT: continuous renal replacement therapy
PICCO: pulse indicator continuous cardiac output
IABP: intra-aortic balloon pump.
